# Unilateral versus bilateral balloon kyphoplasty in treatment of osteoporotic vertebral compression fractures: A randomized controlled trial protocol

**DOI:** 10.1097/MD.0000000000020524

**Published:** 2020-06-19

**Authors:** Sheng Wang, Hui Xu, Wenfei Ni, Qishan Huang, Xiangyang Wang

**Affiliations:** Department of Minimally Invasive Spine Surgery, the Second Affiliated Hospital & Yuying Children's Hospital of Wenzhou Medical University, No.109, Xueyuan Western Road, Wenzhou City, Zhejiang Province, P.R. China.

**Keywords:** bilateral balloon kyphoplasty, osteoporotic vertebral compression fracture, protocol, unilateral balloon kyphoplasty

## Abstract

**Background:**

It is currently controversial whether unilateral or bilateral balloon kyphoplasty (BKP) is superior in terms of postoperative outcomes in treatment of osteoporotic vertebral compression fracture (OVCF). In this context, the aim of this study was to prospectively evaluate and compare the radiographic and clinical outcomes of BKP using unilateral and bilateral approaches.

**Methods:**

This was a randomized controlled study and was approved by the Severance Institutional Review Board in our hospital. The study protocol was designed in accordance with the Declaration of Helsinki guidelines. Patients who complained of chronic back pain secondary to OVCF, which occurred in thoracic lumbar region over 6 months and met the criteria of osteoporosis were the candidates for this procedure. A total of 150 patients were randomized to undergo either unilateral or bipedicular BKP. The outcomes measures inculded pain score, Oswestry Dysfunction Index, compression ratio, kyphotic angle, operation time, and postoperative complications.

**Results:**

We were able to directly compare the outcomes of unilateral versus bilateral BKP and might reveal a better technique in OVCF.

**Trial registration:**

this study protocol was registered in Research Registry (researchregistry5543).

## 1 Introduction

Osteoporosis is a metabolic bone disease characterised by bone loss and bone microstructure changes. It is also the leading cause of vertebral compression fractures, which can result in decreased mobility and quality of life.^[1]^ Osteoporotic vertebral compression fracture (OVCF) is the most common form of osteoporotic fractures, especially in elderly women over the age of 60 years, which are most common in the thoracolumbar spine (T11-L2).^[2]^

Nowadays, there are multiple treatment choices for patients with OVCF, such as conservative treatment, percutaneous vertebroplasty, as well as percutaneous balloon kyphoplasty (BKP). Initial conservative treatment including oral analgesics, bed rest, and physical support were main therapeutic regimen before the application of percutaneous minimally invasive surgery.^[3]^ However, a few patients may still complain of severe pain after conservative treatments and even show the progressive collapse of the vertebral body and kyphosis with or without neurological deficit.^[4]^ BKP is a minimally invasive technique based on percutaneous vertebroplasty. It can provide satisfactory clinical outcomes for the treatment of OVCF, with better efficacy than conservative treatment.

The current standard BKP procedure requires establishment of bilateral puncture channels in the vertebra using a vertebral pedicle approach and the implantation 2balloons.^[5,6]^ Two studies also indicated that traditional bilateral BKP was an effective and safe procedure and had significantly greater benefit than conservative treatment.^[7,8]^ But recently a unilateral BKP has been advocated, reducing the operating time and risks, and increasing the cost-effectiveness of the procedure.^[8]^ Biomechanical study has shown that unilateral kyphoplasty is the same as bilateral kyphoplasty in terms of recovery of vertebral strength, stiffness, and height.^[9]^ Consequently, scholars have attempted to apply unilateral BKP in the treatment of patients with OVCF to obtain better efficacy. Theoretically, compared with bilateral BKP, unilateral BKP involves less radiation exposure and thus less radiation damage, shortens the operating time, and reduces the risk of complications caused by the vertebral pedicle puncture by 50%.^[10]^

Recently, many studies have been published to compare the postoperative results of the 2 techniques.^[11–14]^ However, there is still a lack of high-quality research in the literature. The objective of this study is to conduct a randomized controlled study to compare and analysis the efficacy of unilateral BKP with that of bilateral BKP in the treatment of OVCF. The hypothesis is that the unilateral BKP is more advantageous and superior to bilateral BKP after surgery.

## 2 Materials and methods

### 2.1 Study design

This was a randomized controlled study and was approved by the Severance Institutional Review Board in our hospital. The study protocol was designed in accordance with the Declaration of Helsinki guidelines. The study is also registered in the Research Registry (researchregistry5543).

### 2.2 Patients: recruitment, inclusion, and exclusion criteria

**Figure 1:**
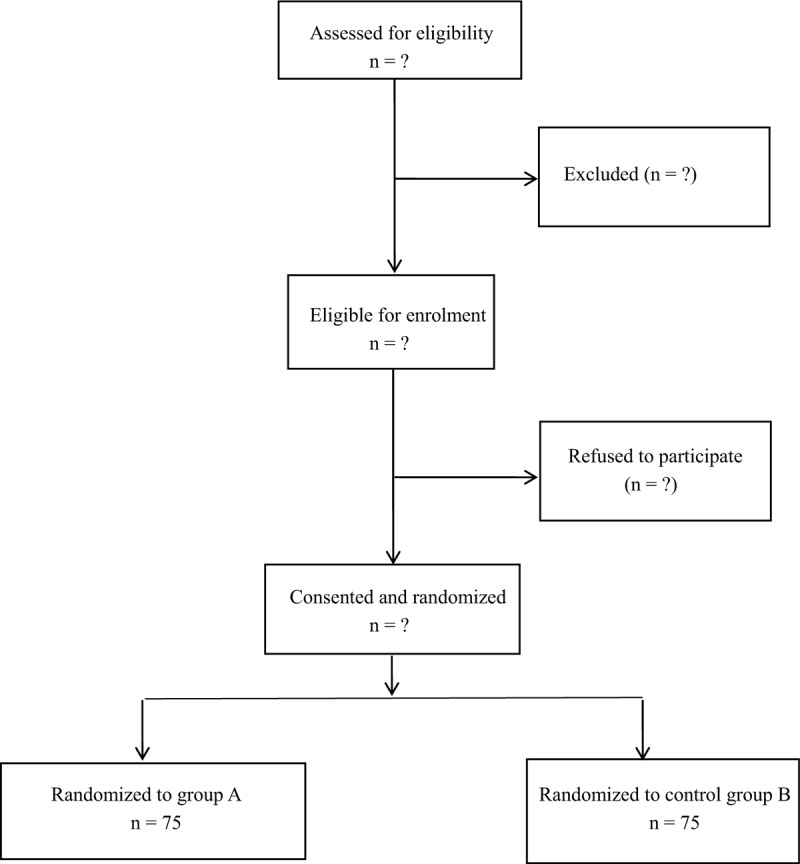
Study design.

Patients who complained of chronic back pain secondary to OVCF, which occurred in thoracic lumbar region over 6 months and met the criteria of osteoporosis were the candidates for this procedure. Before surgery, informed consents were obtained from all patients after a full explanation of the therapeutic procedure (Fig. 1).

The inclusion criteria were as follows:

(1) collapse 15% or more of the vertebral height;
(2) severe back pain related to a single-level OVCF refractory to analgesic medication for at least 2 weeks;
(3) pain score more than 5, measured on a visual analogue scale, and tapping pain at the spinal process of the fractures vertebral body;
(4) using magnetic resonance imaging, the affected vertebral body showed a hypointense signal on T1-weighted images and hyperintense signal on T2-weighted images; and
(5) bone attenuation (T score < -2.5) on bone densitometry.

The exclusion criteria were as follows:

(1) secondary osteoporosis (corticosteroids, endocrine disorders, and an inflammatory process);
(2) inability to give informed consent;
(3) uncorrected coagulopathy;
(4) general poor physical state;
(5) painless OVCF;
(6) spinal metastatic cancer; and neurological symptoms.

### 2.3 Surgical techniques

Patients were placed in the prone position after general anesthesia with tracheal intubation. A 1 cm incision was made lateral to the pedicle of the affected vertebra in those in the unilateral group. A Jamshidi bone biopsy needle was introduced into the pedicle and advanced into the center of the vertebral body at an angle of 30° to 45° relative to the AP axis. A lateral starting point was used for both thoracic and lumbar vertebrae in order reach the midline of the vertebral body while preventing a medial broach through the pedicle. A guide wire was then placed through the Jamshidi needle and the needle removed. A series of dilating cannulae were then advanced over the guide wire until a working cannula was in place. A 15 or 20 mm bone tamp was then introduced into the vertebral body via the cannula and inflated until the balloon was in contact with the subchondral plate, lateral vertebral body wall or anterior cortex of the vertebral body. The balloon was then deflated and removed. Subsequently, cement was injected into the cavity and allowed to harden. Injection was stopped when there was complete filling of the cavity created by the balloon tamp, or if there was a risk of breaching the borders delineated by the tamp. The cannula was then removed and the incision closed. For patients in the bilateral group, the same surgical steps were performed through both pedicles. However, the tips of both cannulae were advanced laterally to the midline and the cement was introduced simultaneously through both cannulae.

In both unilateral and bilateral BKP, bone cement is dispensed into the bone cement syringe after the balloon is expanded. The bone cement is then injected into the vertebra through a working cannula and continuously monitored until a satisfactory filling level is achieved; that is, immediately before the cement exceeds the boundaries of the vertebra. The cannula is removed, the incision is sutured with 1 stitch, and a sterile bandage is applied. Patients were able to walk again after 1 to 2 days.

### 2.4 Postoperative treatment

All patients in both groups were bedridden for 24 hours after surgery. Anti-infection, acid preparation and stomach protection, calcium, and calcitriol were administered on the day of operation. Vital signs, motor sensation of lower extremities, urination, and defecation were observed. After 24 hours of the operation, patients could wear waist brace and walk out of bed under the permissible condition. Simultaneously, oral alendronate sodium (or intramuscular salmon calcitonin) was administered. After hospitalisation for 3 to 5 days, the patients left the hospital. The patients continued to receive anti-osteoporosis treatment after discharge.

### 2.5 Outcome measures

The outcomes measures included pain score, Oswestry Dysfunction Index, compression ratio, kyphotic angle, operation time, and postoperative complications. The compression ratio and kyphotic angle of all fractured vertebrae were measured both before and after surgery. Vertebral height was defined as the endplate-to-endplate distance measured from the anterior aspect of the vertebral body on the lateral radiograph. Standing films were used to measure kyphosis of the fractured vertebral body as the angulation between endplates. Using visual analogue scale scores in the range of 0 to 100 points (0 = no pain; 100 being the most severe pain), the severity of pain was assessed preoperatively and postoperatively within 3 days of the procedure. To evaluate the functional outcome, we also evaluated the Oswestry Disability Index scores preoperatively and at 2 week after the procedure and performed follow-up radiologic studies for approximately 3 months after the procedure in order to rule out the possibility of subsequent vertebral compression fracture.

### 2.6 Statistical analysis

All statistical analyses were performed with the use of SPSS software, version 12 (SPSS Inc., Chicago, IL). Categorical variables were compared by using the χ^2^ and Fisher exact tests and are presented as number and percentage values. Baseline continuous variables were compared by independent 2-sample *t* tests and presented as mean and standard deviation. Paired *t* tests were used to compare the preoperative and postoperative assessments in each group, with *P* < .05 indicating statistical significance. A power analysis was conducted to detect a 5-unit difference in the Oswestry Disability Index between the 2 groups, with the assumption of a 10-unit standard deviation for each group. It was calculated that a minimum of 75 patients per group would be needed to achieve power of 80%.

## 3 Discussion

Among the 750,000 patients with OVCF in the USA, approximately one-third develop chronic pain.^[14]^ Once OVCF occur, 1 in 5 female patients develop new vertebral fractures in the following year.^[15]^ Moreover, kyphosis caused by vertebral fractures negatively affects lung function.^[14]^ Therefore, OVCF treatment should focus on pain relief, vertebral height recovery, and kyphosis improvement to prevent further fractures. Traditionally, treatment for OVCF includes bed rest, narcotic analgesics, braces, and physical therapy. However, these conventional therapies have a negative effect on muscle strength and bone mass, and may lead to serious complications.^[16]^

Over the past decades, vertebroplasty was adopted as an optimal treatment of osteoporotic OVCF, having the advantage of rapid pain relief and long-lasting effect, but it could not restore the decreased height of the vertebral body.^[17–19]^ With the introduction of a newly designed, minimally invasive technique, BKP, the collapsed vertebral body was restored by an inflatable bone tamp and then more viscous polymethylmethacrylate was safely put into the hollow cavity made by the inflatable bone tamp with low pressure.^[12]^ To our knowledge, it is currently controversial whether unilateral or bilateral BKP is superior in terms of postoperative outcomes in treatment of OVCF. In this context, the aim of this study was to prospectively evaluate and compare the radiographic and clinical outcomes of BKP using unilateral and bilateral approaches.

The present study has several limitations. First, the number of patients and the number of compression fractures are limited. Second, dynamic fracture mobility and intraoperative spontaneous reduction were not considered in the current study. Finally, a blind measurement and analysis of vertebral height and local kyphosis were not possible; therefore, bias might influence radiographic outcome.

## Author contributions

**Conceptualization:** Sheng Wang, Hui Xu.

**Data curation:** Sheng Wang, Hui Xu.

**Formal analysis:** Sheng Wang, Hui Xu.

**Funding acquisition:** Wenfei Ni.

**Investigation:** Sheng Wang, Hui Xu, Qishan Huang.

**Methodology:** Sheng Wang, Hui Xu, Qishan Huang.

**Resources:** Xiangyang Wang.

**Software:** Xiangyang Wang.

**Supervision:** Xiangyang Wang.

**Validation:** Sheng Wang, Hui Xu.

**Visualization:** Wenfei Ni.

**Writing – original draft:** Sheng Wang.

**Writing – review & editing:** Xiangyang Wang.
